# Exercise as a potential modulator in anti-Jo-1 positivity: A case report of long-term serological and radiographic stability over 6 years

**DOI:** 10.1097/MD.0000000000049466

**Published:** 2026-06-26

**Authors:** Sha Li, Yanping Liu, Xiaolei Chen, Xiao Zheng

**Affiliations:** a Physical Examination Center, Hebei General Hospital, Shijiazhuang, Hebei, China; b Department of Rheumatology and Immunology, Hebei General Hospital, Shijiazhuang, Hebei, China.

**Keywords:** anti-Jo-1 antibody, antisynthetase syndrome, asymptomatic carrier, case report, interstitial lung disease, natural course

## Abstract

**Rationale:**

The anti-Jo-1 antibody is a hallmark myositis-specific autoantibody strongly associated with antisynthetase syndrome, a multisystem autoimmune disorder characterized by interstitial lung disease, myositis, and arthritis. While its detection typically heralds clinical disease onset, the natural history and optimal management of individuals with isolated anti-Jo-1 positivity who remain asymptomatic remain poorly defined, posing a clinical dilemma regarding monitoring and early intervention.

**Patient concerns:**

A 34-year-old woman presented with incidentally detected anti-Jo-1 antibody positivity during routine screening, remaining entirely asymptomatic throughout the subsequent 6-year observation period.

**Diagnoses:**

Persistent anti-Jo-1 antibody positivity accompanied by stable functional and imaging results, consistent with an asymptomatic, non‑progressive carrier state of anti‑Jo‑1 antibody positivity.

**Interventions:**

Given the absence of clinical symptoms or objective evidence of systemic inflammation, a strategy of regular surveillance was adopted without initiating immunosuppressive therapy. The patient was encouraged to maintain her regular lifestyle, including at least 150 minutes of moderate-intensity aerobic exercise per week. She underwent clinical and serological assessment every 6 to 12 months and annual thoracic high-resolution computed tomography over a 6-year follow-up period.

**Outcomes:**

The patient remained entirely asymptomatic throughout 6 years of observation. Serial high-resolution computed tomography scans demonstrated no radiological progression, with stable nodules and no emergence of new findings suggestive of interstitial lung disease, such as reticulation, honeycombing, or consolidation. Inflammatory markers consistently remained within normal ranges.

**Lessons:**

This case reinforces that anti-Jo-1 positivity does not invariably lead to clinical disease and supports a conservative, surveillance-based approach in asymptomatic carriers. The potential immunomodulatory role of sustained physical activity highlights the importance of integrating lifestyle assessment into management, while further research is needed to stratify risk and guide intervention timing.

## 1. Introduction

Antisynthetase syndrome (ASS) is an autoimmune disease. It is defined by autoantibodies that target aminoacyl-tRNA synthetases. Among these, anti-Jo-1 antibody is the most common. Typical symptoms of ASS include interstitial lung disease (ILD), muscle inflammation (polymyositis/dermatomyositis), fever, mechanic’s hands, and joint inflammation (arthritis).^[1]^

Usually, having these autoantibodies means there is a risk of organ damage, especially progressive ILD. However, how the disease appears in patients can vary greatly^[2]^ current research mainly studies patients with clear symptoms. But for people who only have the antibodies without symptoms, many questions remain.^[3]^ These include their long-term outlook, risk of the disease worsening, and how they should be managed.

This case report describes a patient with anti-Jo-1 antibodies. This patient stayed clinically and radiologically stable for a long time. By reporting this case, we hope to start a discussion. We want to discuss how to define such “asymptomatic carrier states,” understand their natural course, and decide if and how they need clinical monitoring.

## 2. Case report

Six years ago, a 34-year-old female presented to our hospital following the incidental identification of anti-Jo-1 antibody positivity during a routine physical examination. Her past health were all normal. The patient reported no symptoms such as cough, dyspnea, fever, myalgia, arthralgia, skin rash, or Raynaud’s phenomenon. Vital signs were normal. Cardiopulmonary, musculoskeletal, dermatologic, and neurologic examinations revealed no abnormalities. Findings were consistent with a previously healthy individual. The patient reported maintaining a regular lifestyle, including a balanced diet, adequate sleep, and at least 150 minutes of moderate-intensity aerobic exercise per week. This active lifestyle was encouraged as part of her non-pharmacological management plan.

The patient had no chronic medical conditions, surgical history, or significant infections, with no personal history of cancer or metabolic disorders. Her family history was negative for autoimmune diseases, ILD, myopathies, and early-onset cardiovascular conditions. She reported no psychiatric illness, substance use, or documented genetic disorders.

Initial systematic evaluation revealed a positive result for anti-Jo-1 antibody. The antinuclear antibody was weakly positive at 1:100. This was measured using indirect immunofluorescence. The results of the myositis antibody panel (including anti–PL-7, PL-12, EJ, OJ, SRP, and MDA5 antibodies) and the anti‑extractable nuclear antigen panel (including anti-Ro‑52, Sm, AHA, AnuA, Scl‑70, PM‑Scl, RNP, SSA, and SSB antibodies) were all negative. Anti–double‑stranded DNA antibody, rheumatoid factor, anti‑cyclic citrullinated peptide antibody, anti-keratin antibody, and anti‑streptolysin O also tested negative. Immunoglobulin (A, G, M) and complement (C3, C4, C1q) levels were within normal limits. Inflammatory markers, including absolute lymphocyte subset counts, creatine kinase, aldolase, erythrocyte sedimentation rate, procalcitonin, C‑reactive protein, interleukins (1β, 2, 4, 6, 8, 10, 12p70, 17), tumor necrosis factor‑α, and interferons (α, γ), were all within normal ranges. Liver and kidney function tests, as well as tumor markers such as α‑fetoprotein, carcinoembryonic antigen, and cancer antigen 125, showed no abnormalities.

To evaluate potential pulmonary involvement, the patient underwent high-resolution computed tomography (HRCT) of the chest. Baseline scanning demonstrated only a few stable tiny solid nodules, which did not meet the diagnostic criteria for typical interstitial pneumonia.

**Figure 1.**
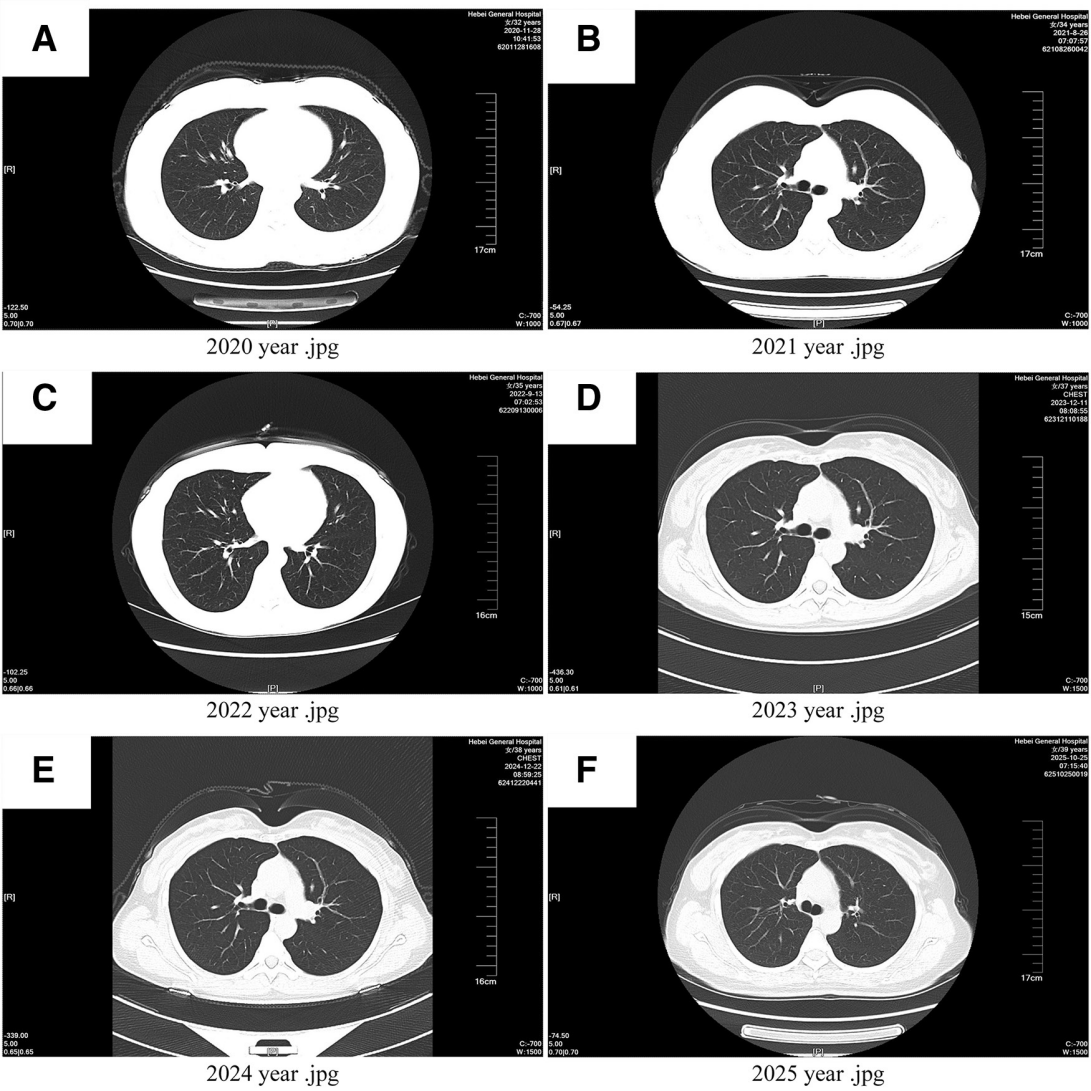
Serial chest CT images of the anti-Jo-1 antibody-positive patient from 2020 to 2025 (A–F). A few stable tiny solid nodules are visible, with no reticular opacities, honeycombing, or consolidation observed.

During the subsequent 6 years of follow-up, the patient underwent clinical assessments and hematological examinations every 6 to 12 months (including the myositis antibody panel, antinuclear antibody profile, immunoglobulins, complement, and other inflammatory markers), with annual repeat HRCT of the chest. Serial evaluations confirmed the persistent absence of clinical symptoms and maintenance of normal inflammatory indices. Comparative review of 6 years of sequential CT images by radiologists verified no definitive progression of the previously observed minimal changes, and no new signs indicative of active ILD (such as reticular opacities, honeycombing, or consolidation) were identified (Fig. 1). The sequence and results of these evaluations over the 6-year period are summarized in Table 1.

**Table 1:** Timeline of clinical, serological, and radiological assessments over 6 years of follow-up.

Year	Assessments performed	Key findings
Baseline (Year 0)	HRCT, serology (anti-Jo-1, ANA, myositis panel, inflammatory markers)	Anti-Jo-1 positive, ANA 1:100, all other labs normal. HRCT: few stable tiny nodules
Year 1–5 (every 6–12 mo)	Clinical exam, serology (inflammatory markers, autoantibodies)	Asymptomatic. All serological markers within normal ranges
Year 6	HRCT, PFTs, lower limb MRI, complete serology	Asymptomatic. HRCT unchanged (no ILD signs). PFTs and MRI normal. Anti-Jo-1 persistently positive

ANA = antinuclear antibody, HRCT = high-resolution computed tomography, MRI = magnetic resonance imaging, PFTs = pulmonary function tests.

**Figure 2.**
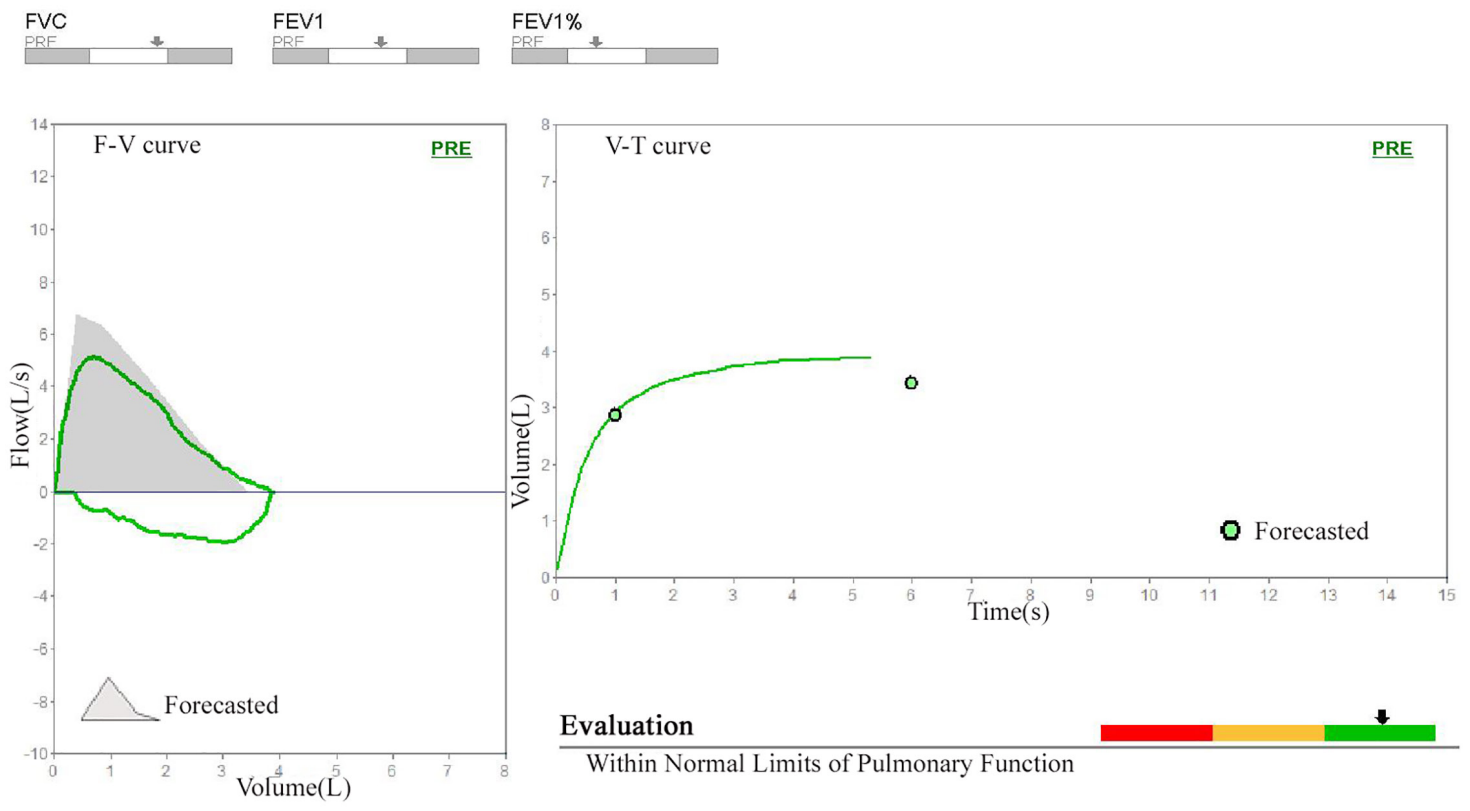
Results of pulmonary function tests: The flow-volume curve and volume-time curve showed that the patient’s pulmonary function was normal.

**Figure 3.**
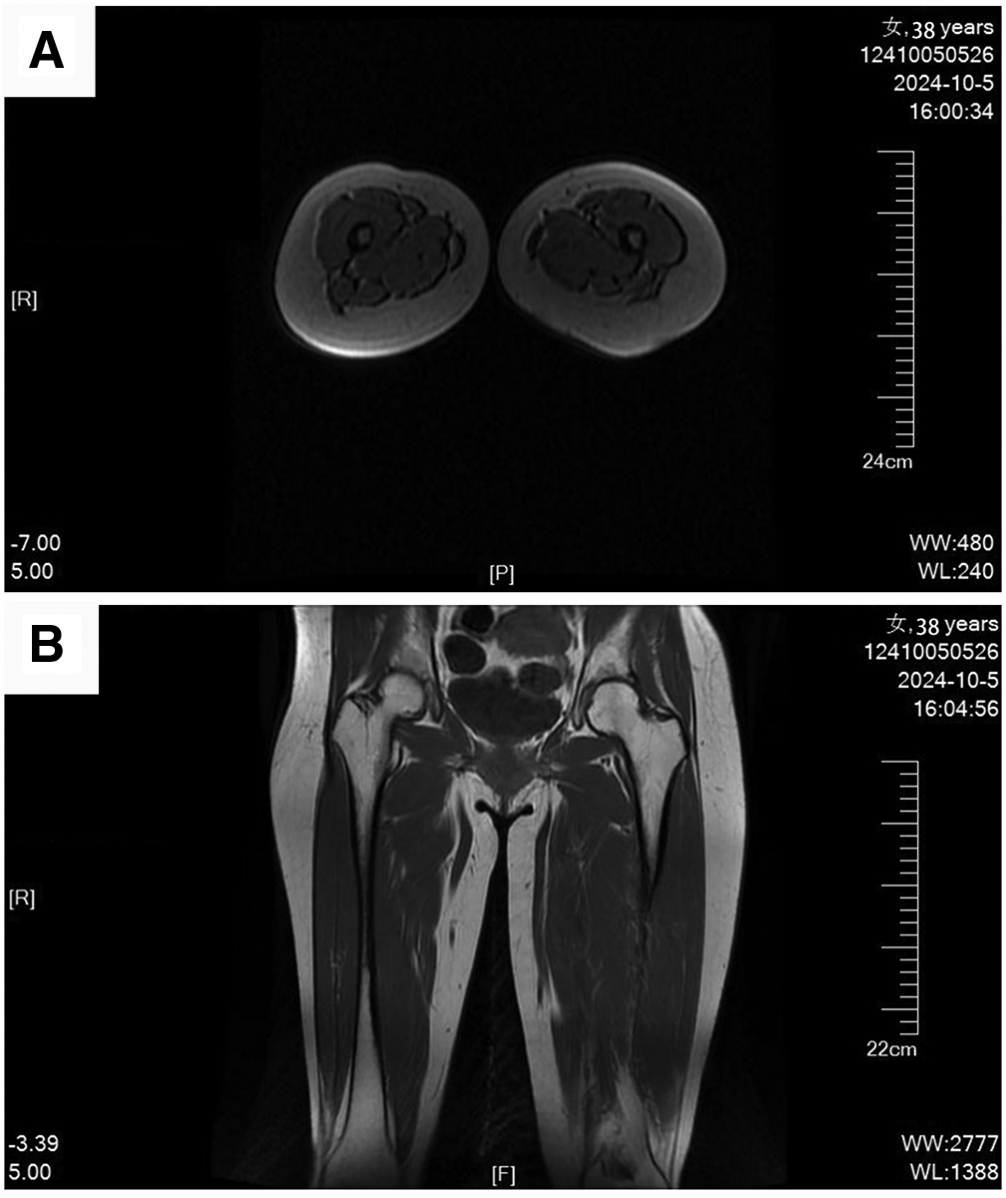
Results of plain MRI of both lower extremities were normal; no abnormal signal shadows indicating skin or muscle swelling were observed. (A) Transverse plane. (B) Coronal plane. MRI = magnetic resonance imaging.

Recently, to further assess the patient’s condition, pulmonary function tests (including lung volumes and diffusing capacity) were performed, with results completely normal (Fig. 2). Magnetic resonance imaging (MRI) of both lower limbs revealed no abnormal signals indicative of skin or muscle swelling (Fig. 3). Persistent anti-Jo-1 antibody positivity with stable functional and imaging findings, consistent with an asymptomatic, nonprogressive carrier state of anti-Jo-1 antibody positivity. In the absence of clinical symptoms and objective evidence of inflammation, a regular monitoring strategy was implemented, which included encouraging at least 150 minutes of moderate-intensity aerobic exercise per week.

## 3. Patient perspective

The patient fully supports the conservative monitoring strategy and has consented to the public reporting of this case to promote greater attention within the medical community to this type of long-term asymptomatic antibody carrier state. She expressed satisfaction with the current follow-up plan, noting that this noninvasive management approach allows her to maintain a normal lifestyle while avoiding the burden of overtreatment. The stable clinical and imaging outcomes over the past 6 years have provided her with substantial reassurance, and throughout the observation period, she has remained well-informed and actively involved in decision-making regarding her care. The patient also hopes that this real-world case can serve as a reference for subsequent research, contributing to the exploration of more scientific and individualized clinical management pathways.

## 4. Discussion

This case presents an unusual clinical scenario: persistent anti-Jo-1 antibody positivity coexisting with long-term clinical and radiological stability. This is relatively rare within the spectrum of ASS, which is characterized by multisystem involvement and potential progression.^[4]^

Firstly, this case challenges the assumption that anti-Jo-1 antibody positivity necessarily predicts imminent clinical onset.^[5]^ It suggests the existence of a “preclinical” or “subclinical” state in which autoimmune serological abnormalities may precede tissue damage by several years or longer. The recently performed pulmonary function tests (including lung volumes and diffusing capacity), which yielded completely normal results, along with the absence of abnormal signals on MRI of both lower limbs, further reinforce the notion that the patient is in a truly nonprogressive, preclinical carrier state, with no objective evidence of subclinical pulmonary impairment or myositis. The stable condition observed may be attributed to multiple factors, including genetic background, absence of environmental triggers, effective immune regulation, or functional differences in the antibody itself.^[6]^ Notably, the patient maintained a regular exercise regimen of at least 150 minutes of moderate-intensity aerobic activity per week. Exercise is known to have anti-inflammatory and immunomodulatory effects, such as promoting the release of anti-inflammatory cytokines (e.g., IL-10), reducing systemic inflammation, and enhancing regulatory T cell function.^[7]^ While we cannot establish causality, it is plausible that her sustained physical activity contributed to maintaining immune homeostasis and preventing subclinical inflammation from progressing to overt disease. This observation aligns with growing evidence that lifestyle interventions, including regular exercise, may modulate disease activity in autoimmune conditions.^[8]^

Second, this case raises practical considerations regarding clinical management. For such patients, overly aggressive immunosuppressive therapy may pose unnecessary risks. Current treatment strategies typically prioritize intervention based on the presence of symptoms and objective findings, such as declining pulmonary function or radiological progression.^[9]^ This case supports a rationale for adopting a cautious monitoring approach in patients who remain asymptomatic, exhibit no functional impairment, and demonstrate stable imaging findings. The inclusion of pulmonary function tests and muscle imaging (e.g., MRI) in the initial and follow-up assessments, as demonstrated in this case, provides a more comprehensive evaluation beyond HRCT alone, aiding in the decision for continued surveillance. However, the optimal frequency of monitoring and the selection of appropriate tools – including the balance between pulmonary function tests and computed tomography scans – require further determination through larger cohort studies.

Finally, this case underscores the broad clinical spectrum of ASS. Future research should aim to identify biomarkers or clinical characteristics that can predict disease transition from asymptomatic to symptomatic states, thereby enabling risk stratification and personalized management.^[10]^

## 5. Strengths and limitations

### 5.1. Strengths

This report offers a rare, well-documented longitudinal observation of an isolated anti-Jo-1 antibody carrier who remained clinically and radiologically stable over 6 years, challenging the traditional serological–clinical correlation in ASS.^[11]^ The consistent use of annual HRCT and serial inflammatory marker assessments provides objective evidence supporting the existence of a nonprogressive, preclinical state. Furthermore, the recently performed pulmonary function tests (with completely normal results) and MRI of both lower limbs (showing no abnormal signals) provide additional evidence against subclinical pulmonary impairment or myositis, strengthening the conclusion of a benign carrier state. Importantly, this case aligns with emerging immunophenotypic concepts in autoimmune diseases, where certain autoantibody-positive individuals may never progress to overt clinical disease due to immunological tolerance or regulatory mechanisms – a phenomenon increasingly recognized in other antibody-associated disorders such as antinuclear antibody-positive healthy cohorts.^[12]^ The management approach described – active surveillance without preemptive immunosuppression – reflects a growing clinical emphasis on risk-stratified, symptom-driven treatment, consistent with recent guidelines for myositis-associated ILD.^[9]^

Notably, the authors plan to conduct annual follow-up evaluations of this patient, encompassing serial serological and imaging assessments. These longitudinal data will be regularly shared with the scientific community through publications and academic exchanges, aiming to foster collaboration with experts and scholars in the field, thereby advancing the collective understanding of the natural history and pathobiology associated with anti-Jo-1 antibody carriers.

### 5.2. Limitations

As a single-case report, the findings cannot be generalized, and the possibility of eventual disease progression beyond the 6-year follow-up period cannot be excluded. The patient’s stable phenotype may be influenced by unmeasured genetic, epigenetic, or environmental factors, such as her regular exercise regimen, which has known immunomodulatory effects but was not systematically evaluated. Furthermore, while the patient reported a balanced diet and adequate sleep, these lifestyle factors were not systematically quantified or controlled for, and their potential synergistic or independent effects on immune modulation remain unknown. Additionally, the study lacks advanced immunoprofiling data – such as autoantibody subclass, functional assays, or cytokine/chemokine profiling – that could help elucidate the mechanisms underlying her nonprogressive state.^[13]^ While comprehensive functional assessments (pulmonary function tests and muscle MRI) were performed, longer-term follow-up and larger cohort studies are needed to validate these observations and establish evidence-based monitoring protocols.

## 6. Conclusion

This case presents a 6-year observation of an asymptomatic patient with persistent anti-Jo-1 antibody positivity, demonstrating sustained stability in both serological parameters and radiographic features without progression to antisynthetase syndrome. Of note, the patient’s adherence to a regular moderate-intensity exercise regimen may have contributed to maintaining immune homeostasis. These observations provide a clinical reference supporting a conservative management strategy centered on periodic monitoring for such asymptomatic antibody carriers, with lifestyle assessment integrated into the comprehensive evaluation. Furthermore, this study highlights the need for future exploratory research to identify predictors of disease progression and to systematically evaluate the immunomodulatory effects and clinical value of exercise interventions in similar populations.

### 6.1. “Take-away” lesson

Isolated anti-Jo-1 positivity can persist as a long-term, nonprogressive carrier state, decoupling seropositivity from clinical disease. Asymptomatic patients with stable imaging/function should be managed with active surveillance rather than preemptive immunosuppression. Lifestyle factors such as regular exercise may modulate immune activity, though this warrants systematic investigation. Future research should focus on identifying predictors of progression and evaluating lifestyle-based strategies in disease modulation.

## Acknowledgments

All authors made a substantive intellectual contribution, read and approved the final version of the manuscript and agreed to be accountable for all aspects of the work.

## Author contributions

**Funding acquisition:** Sha Li.

**Investigation:** Sha Li, Yanping Liu, Xiaolei Chen, Xiao Zheng.

**Software:** Sha Li, Xiaolei Chen.

**Writing – original draft:** Sha Li.

**Writing – review & editing:** Sha Li.
